# The Genomic Profile of Pregnancy-Associated Breast Cancer: A Systematic Review

**DOI:** 10.3389/fonc.2020.01773

**Published:** 2020-09-11

**Authors:** Anna-Maria Korakiti, Myrto Moutafi, Eleni Zografos, Meletios-Athanasios Dimopoulos, Flora Zagouri

**Affiliations:** Department of Clinical Therapeutics, Alexandra Hospital, School of Medicine, National and Kapodistrian University of Athens, Athens, Greece

**Keywords:** pregnancy, breast, cancer, gene, mutation

## Abstract

Breast cancer is the most common malignancy diagnosed during pregnancy. Strong data on the genomic profile of pregnancy-associated breast cancer are lacking. This systematic review aims to integrate and analyze all existing data from the literature regarding the genomic background and the gene mutational patterns of pregnancy-associated breast cancer. Using various genomic analysis methods, multiple differentially expressed genes and numerous non-silent mutations have been detected. More particularly, our review demonstrates the aberrant expression of several oncogenes (e.g., *MYC, SRC, FOS*), tumor suppressor genes (e.g., *TP53, PTEN, CAV1*), apoptosis regulators (e.g., *PDCD4, BCL2, BIRC5*), transcription regulators (e.g., *JUN, KLF1, SP110*), genes involved in DNA repair mechanisms (e.g., Sig20, *BRCA1, BRCA2, FEN1*), in cell proliferation (e.g., *AURKA, MKI67*), in the immune response (e.g., *PD1, PDL1*), and in other significant biological processes (e.g., protein modification, internal cell motility). Further research on the genomic profile of pregnancy-associated breast cancer is urgently required in order to identify potential biomarkers facilitating early-stage diagnosis and individualized therapy.

## Introduction

Breast cancer is the most common type of malignancy diagnosed in women. Its incidence is notably rising with increasing age (1, 2). Breast cancer represents a heterogeneous disease with fundamental histological variations among patients of different age, sex, and in certain conditions such as gestation. Pregnancy-associated breast cancer (PABC) is generally defined as breast cancer diagnosed anytime during gestation, lactation or within 1 year after delivery (2–4). Several other PABC definitions with minor modifications regarding the postpartum period exist in the literature (2, 5). Along with melanoma and cervical cancer, they are the most frequent types of pregnancy related cancer (3, 6, 7). Every year, 1 in 3,000–10,000 women is diagnosed with breast cancer during pregnancy, representing only 0.2–3.8% of overall breast cancer cases (3, 4). As women postpone childbearing to a later age in our society, PABC rate is expected to increase significantly (2, 3).

PABC management consists a real challenge for physicians as both the mother and the fetus may be critically damaged (3, 6). Due to the rarity of the disease, strong data regarding PABC treatment are lacking and current guidelines are based on small retrospective studies and systematic meta-analyses. Cancer diagnosis in a period of hope and joy is an unendurable situation that may trigger symptoms of psychological distress such as depression, anxiety, social isolation and self-blame. On the one hand, patients face a life-threatening disease and an uncertain pregnancy. On the other hand, medical professionals face an ethical dilemma involving the future mother and her unborn child; what is best for the mother in terms of aggressive chemotherapy may be fatal for the fetus and vice versa, delaying therapy and protecting the fetus may have a negative impact on the mother as the tumor progresses (8).

The molecular nature of PABC remains an unknown field and considerable controversy exists in the literature regarding the influence of pregnancy on breast cancer prognosis (3). PABC exhibits particularly aggressive behavior and its poor outcome is largely attributed to tumor characteristics; advanced T stage in diagnosis, nodal involvement, high histologic grade, negative estrogen (ER) and progesterone (PR) status and *HER-2* overexpression (4, 9). Despite the substantial efforts in managing breast cancer during pregnancy, yet there has been little progress in explaining PABC biological characteristics.

This review aims to synthesize all existing data from the literature regarding gene expression in PABC. Genomic profiling studies identify both the spectrum of somatic mutational patterns and the genomic heterogeneity of the disease. A deeper understanding of PABC underlying mechanism may potentially explain its rather aggressive clinical behavior and may lead to individualized therapies.

## Methods

**Figure 1:**
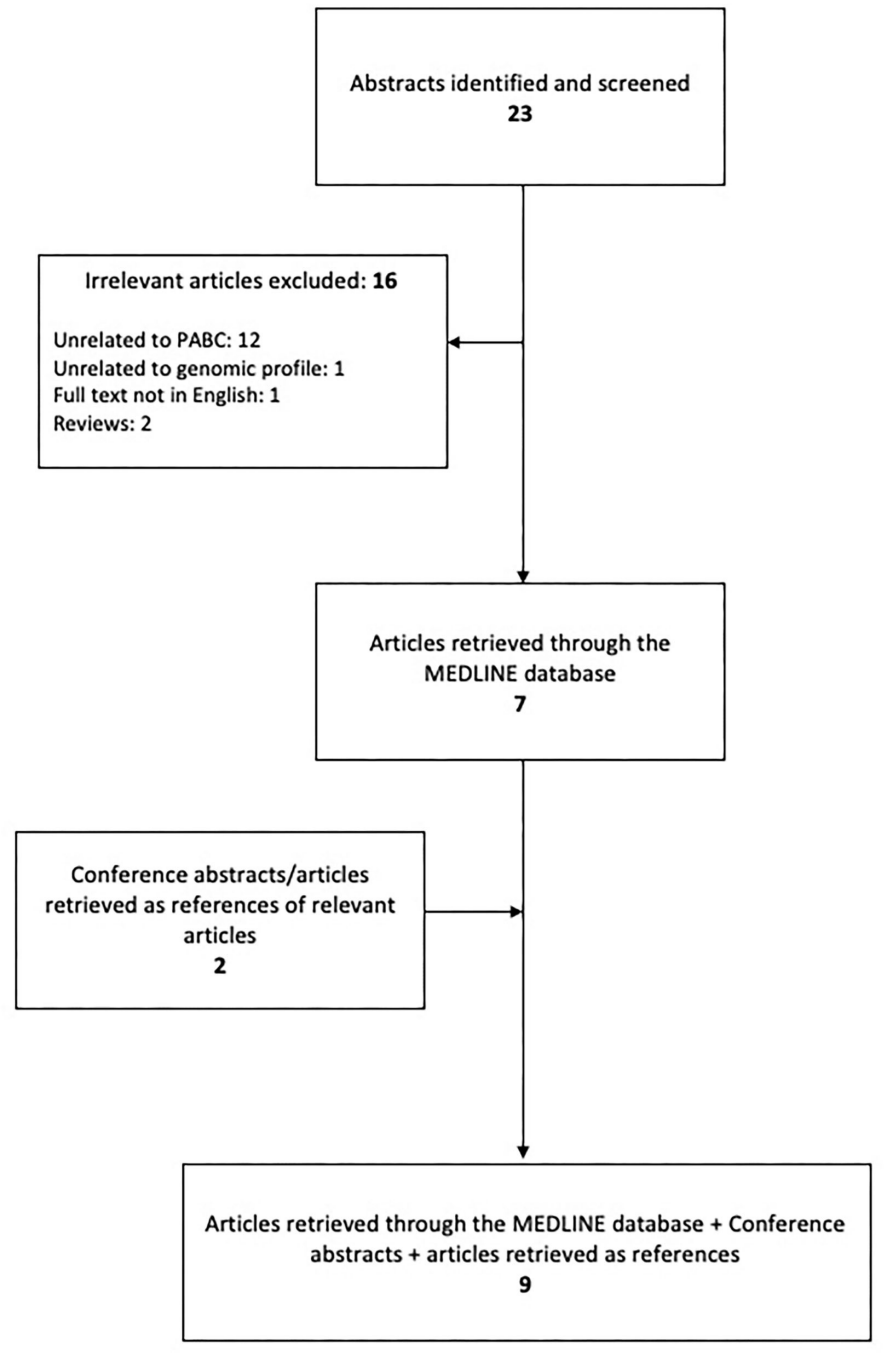
Stages of the search strategy.

All eligible articles included in this literature review were identified in the Medline/PubMed bibliographical database and the research was conducted according to the PRISMA guidelines (10); the end-of-search date was June 10, 2020. The search strategy consisted of the following keywords: [breast AND (neoplasm OR neoplasms OR cancer OR cancers OR carcinoma OR carcinomas)] AND (pregnancy OR pregnant OR gestation) AND (genomics OR genomic OR gene OR genes OR mutation OR mutations). Furthermore, in order to identify any additional eligible articles, reference lists were also meticulously examined resulting in a total of 9 articles to be included as shown in Figure 1.

While working separately, two researchers (AMK and MM) searched the literature and another pair of investigators (AMK and EZ) independently extracted data from each eligible study. In case of disagreement between the members of each pair, team consensus was obtained after consulting the principal designers of the study (FZ and MAD). The articles included in this systematic review had to meet certain inclusion criteria: (1) studies highlighting the genomic profile of PABC, including mutational patterns, (2) studies based on the analysis of biological samples and/or bioinformatic approaches or computational algorithms with data originating from databases, (3) articles written in the English language. Publications were excluded if they met one or more of the following criteria: (1) animal studies without subsequent validation in human specimens, (2) reviews of literature, comments, letters or duplicate publications.

## Results

The search strategy retrieved 23 articles. Of these, 16 were omitted based on the exclusion criteria and 7 were eligible (11–17). While examining the references of eligible articles, 2 more articles were included (18, 19). A summary of the studies describing the genomic profile of pregnancy-associated breast cancer is demonstrated in Table 1.

**Table 1:** Summary of studies describing the genomic profile of pregnancy-associated breast cancer.

**References**	**Sample**	**Technique**	**Groups**	**Up-regulated DEGs in PABC**	**Down-regulated DEGs in PABC**	**PABC enriched mutations**
Nguyen et al. (11)	FFPE	WGS Microarray assay	54 PABC -vs-113 non-PABC	MYC^*^	TP53^*^	TP53^*^, PIK3CA^*^ Mucin gene family Sig1, Sig20
Azim et al. (12)	FFPE	Multiplex PCR Microarray assay	54 PABC -vs-113 non-PABC	PD1, PDL1, BRCA1, Gene sets related to SRC, IGF1, β-catenin GPCR pathway Serotonin receptor pathway	AKTmTOR	TP53^*^, PIK3CA^*^
Walter et al. (18)	FFPE	RT-PCR IHC	25 PABC -vs.-adjacent normal breast tissue		PTEN, PDCD4, BCL2	
Shen et al. (13)	FFPE	PCR-based LOH assay	12 PABC -vs-15 non-PABC		BRCA2	
Johansson et al. (17)	N/A	N/A	14 sporadic PABC -vs-10 hereditary PABC			BRCA1
Harvell et al. (14)	Fresh frozen tissue & FFPE	LCM Microarray assay	6 PABC epithelia -vs-7 normal adjacent epithelia	MKI6, BIRC5, MMP11, RRM2, PRC1, MKI67, KIF2C, AURKA	PTEN, CAV1, GAS1, p63, Ribosomal genes	
Harvell et al. (14)	Fresh frozen tissue & FFPE	LCM Microarray assay	6 PABC stroma -vs-4 non-PABC stroma		HOX genes ECM-encoding genes	
Zhang et al. (15)	Microarray profile datasets (12, 14)	Bioinformatic analysis	74 PABC -vs −126 non PABC	KLHL3, MMP9, TRIM69, ASB6, PXN	CREB1, ARF3, UBA5, SIAH1, HECTD1, MEX3C, UBE2Q2, FBXO22, EIF4A3,	
Zhou et al. (16)	Microarray profile dataset (14)	Bioinformatic analysis	7 PABC stroma -vs-4 normal adjacent stroma	JUN, FOS, MYC, ACTA2, IL18, CD274		
Thanmalagan et al. (19)	Microarray profile dataset (14)	Bioinformatic analysis	20 PABC -vs −13 non PABC	KLF1, FEN1, SP110, MUC1		

*PABC, pregnancy-associated breast cancer, FFPE, formalin-fixed paraffin embedded tissue, WGS, whole genome sequencing, PCR, polymerase chain reaction, RT-PCR, real time PCR, IHC, immunohistochemistry, LOH, loss of heterozygosity, LCM, laser capture microdissection*.

* *Up-regulated/Down-regulated/Mutationally enriched: when compared to normal tissue (no statistically significant difference between PABC and non-PABC groups)*.

As far as the genomic analysis approach is concerned, four studies were based on formalin-fixed paraffin embedded (FFPE) tissue analysis (11–13, 18), one study examined fresh frozen tissue (14), three studies were associated with bioinformatic analysis and microarray profile datasets (15, 16, 19), and one study did not provide precise information regarding the methodology steps (17). Importantly, all studies included in the review were case-control studies; thus, the groups compared in each study exhibited similar clinicopathological characteristics.

According to the literature, the most frequently up-regulated genes encountered in PABC are several oncogenes: *MYC* (11, 16), *FOS* (16), *MUC1* (19), and gene sets related to *SRC* (12); multiple apoptosis regulators: *BIRC5* (14), *TRIM69* (15); transcription regulators: *JUN* (16), *KLF1, SP110* (19); genes involved in the immune response: *PD1, PDL1* (12), *IL18, CD274* (16); in DNA repair mechanisms: *BRCA1* (12), *FEN1* (19); in DNA replication: *RRM2* (14); in cellular growth and proliferation: *IGF1* (12), *MKI6, PRC1, MKI67, KIF2C, AURKA* (14); in protein modification: *KLHL3, ASB6* (15); in collagen degradation: *MMP11* (14), *MMP9* (15); in cell adhesion: β*-catenin* (12), *PXN* (15); and in internal cell motility: *ACTA2* (16). Additionally, high expression of the G-protein coupled receptor (GPCR) pathway and the serotonin receptor pathway is demonstrated (12).

In contrast, the most commonly down-regulated genes detected in PABC are numerous tumor suppressor genes: *TP53* (11), *PTEN* (14, 18), *CAV1* (14); cell cycle regulators: *AKTmTOR* (12), *GAS1* (14); apoptosis regulators: *PDCD4, BCL2* (18), *p63* (14), *SIAH1* (15); transcription regulators: *HOX* genes (14), *CREB1* (15); ribosomal genes (14); ECM-encoding genes (14); genes involved in DNA repair mechanisms: *BRCA2* (13); in protein modification: *UBA5, HECTD1, MEX3C, UBE2Q2, FBXO22* (15); in protein transport: *ARF3* (15); and in mRNA processing: *EIF4A3* (15).

To conclude, non-silent mutations characterizing PABC are most frequently enriched in the tumor suppressor gene *TP53* and the cell cycle regulator *PIK3CA*, the mucin gene family involved in glycosylation (*MUC17, MUC2, MUC4, MUC12, MUC20*) and the *BRCA1* gene (11, 12, 17). Sig1 and Sig20 are the most common single-base alterations highlighted in PABC patients (11). A more detailed analysis of the results is presented in the following discussion.

## Discussion

This review aims to systematically summarize all existing data from the literature regarding the molecular nature of pregnancy-associated breast cancer. Multiple differentially expressed genes and numerous non-silent mutations have been detected. More particularly, our review demonstrates the aberrant expression of several oncogenes (e.g., *MYC, SRC, FOS*), tumor suppressor genes (e.g., *TP53, PTEN, CAV1*), apoptosis regulators (e.g., *PDCD4, BCL2, BIRC5*), transcription regulators (e.g., *JUN, KLF1, SP110*), genes involved in DNA repair mechanisms (e.g., Sig20, *BRCA1, BRCA2, FEN1*), in cell proliferation (e.g., *AURKA, MKI67*), in the immune response (e.g., *PD1, PDL1*) and in other significant biological processes (e.g., protein modification, internal cell motility). The most significant studies on the genomic profile of PABC are shortly presented.

In one of the most recent studies, Nguyen et al. analyzed retrospectively 167 breast cancer patients, 54 of whom were diagnosed during pregnancy, in order to identify specific molecular alterations characterizing PABC (11). No significant differences were found among PABC and non-PABC subgroups in terms of the copy number alteration (CNA) profiles. Of note, *MYC* oncogene was the most commonly amplified, whereas *TP53* tumor suppressor gene was the most frequently deleted gene in both subgroups. The study demonstrated that PABC group had a significantly higher number of non-silent mutations. Across the whole cohort, *TP53* and *PIK3CA* were the most frequently mutated genes. PABC group though was associated with a higher frequency of mutations in the mucin gene family that plays a major role in the mechanism of glycosylation (*MUC17, MUC2, MUC4, MUC12*, and *MUC20*); of note, alterations in the biological functions of glycosylation are correlated with breast carcinogenesis and metastasis (20). While investigating particular patterns of mutations on cancer genomes termed signatures, the researchers proved that the base-substitution mutational Signature 1 (Sig1) and Signature 20 (Sig20) predominated in PABC subgroup. As it is well established in the literature, Sig1 is associated with age at diagnosis. Sig20 was proven to be related to DNA mismatch repair (MMR) deficiency due to copy number loss of *MSH2* allele (21). In addition, Sig-20-positive patients were highly associated with PR negative status and a shorter disease-free survival (DFS) rate when compared to Sig20-negative patients.

Azim et al. also evaluated the biological pathways of PABC aiming to define the prognostic value of the different molecular aberrations (12). Even though the study found no significant differences in somatic mutations among pregnant and non-pregnant breast cancer patients, *TP53* and *PIK3CA* were the most commonly mutated genes in both subgroups, similarly to the aforementioned study. Moreover, Azim et al. demonstrated that the expression of two particular pathways was significantly enriched in breast tumors diagnosed during pregnancy when compared to non-pregnancy related cases; the G-protein coupled receptor pathway (GPCR) and the serotonin receptor signaling pathway (22, 23). Using transcriptomic profiling methods, the study also revealed that PABC tumors had a higher expression of *PD1, PDL1, BRCA1*, and gene sets related to *SRC, IGF1* and β*-catenin* and a lower expression of the *AKTmTOR* gene set. None of the above differentially expressed genes (DEGs) was statistically associated with DFS in the multivariate model.

A few years ago, Walter et al. focused on the expression of the tumor suppressor gene *PTEN* and on the levels of the apoptosis regulators *PDCD4* and *BCL2* in PABC, and on their role as potential markers of poor prognosis (18). In the analysis, protein levels of the aforementioned genes were lower in PABC tumors when compared to adjacent normal breast tissue. A statistically significant correlation was found in PABC group between *PTEN* gene downregulation and *miR-21* overexpression. Overexpression of *miR-21* was demonstrated in the PABC subgroup and it was correlated positively with lymph node involvement and negatively with prognosis.

Two studies regarding *BRCA1* and *BRCA2* mutations were retrieved from the literature. On the one hand, Shen et al. studied retrospectively 12 archival samples from PABC patients and demonstrated high frequency of loss of heterozygosity (LOH) at the *BRCA2* gene when compared to non-PABC cases, suggesting an initial genetic event in the pathogenesis of PABC (13). On the other hand, Johansson et al. investigated the influence of pregnancy on the risk of developing breast cancer in *BRCA1* and *BRCA2* mutation carriers (17). The statistical analysis proved that more women with *BRCA1* mutations developed PABC and implied a close monitoring of women with *BRCA1* familial mutations during and after pregnancy.

One of the largest studies on the genomic signatures of PABC was conducted by Harvell et al. who meticulously examined breast epithelial and stromal cells gene regulation by estrogens and progesterone (14). Both epithelia and tumor-associated stroma of PABC were characterized by enhanced expression of genes related to the immune response and the cell cycle regulation, many of which were hormone regulated. Tumor microenvironment influenced by the several-fold increased gestational hormones had a pivotal role in tumor aggressiveness (24, 25). In addition, the study revealed decreased expression of extracellular matrix (ECM)-encoding genes in PABC-associated stroma that is correlated with cancer invasion and metastasis (26).

Zhang et al. recently published an analysis on core genes and their clinical roles in PABC. Their research was based on two microarray profile datasets that derived from studies previously described in our review (12, 14), but instead focused on the identification of molecular biomarkers using the collective data (15). A total of 239 DEGs were detected in PABC, including 101 up-regulated and 138 down-regulated genes. The up-regulated DEGs were mainly enriched in the immune response, the fatty acid activation and the fibroblast growth factor signaling pathway, whereas the down-regulated DEGs were primarily involved in the activation of DNA fragmentation factor and the apoptosis-induced DNA fragmentation. The 14 most significant identified node degree genes given by the number of links in the protein interaction network were the following: *CREB1, ARF3, UBA5, SIAH1, KLHL3, HECTD1, MMP9, TRIM69, MEX3C, ASB6, UBE2Q2, FBXO22, EIF4A3, PXN*. The node degree can be used to define groups of genes that are co-regulated and consequently may serve similar functions. Interestingly, the up regulation of *ASB6* was the only highly associated with worse overall survival (OS) rate in PABC, particularly in triple negative molecular subtype and pre-menopausal status. The researchers indicated that *ASB6* may have an essential role as a prognostic biomarker and a therapeutic target in PABC management (27).

Zhou et al. also investigated the genomic pathways of PABC through bioinformatic analysis of a microarray dataset (14). This study was differentiated by detecting DEGs in tumor-associated stroma of PABC (16). A total of 480 DEGs were identified among tumor-related and normal stromal cells in PABC patients. The node degree genes *JUN, FOS, MYC* and *ACTA2*, including the up-regulated DEGs *IL18* and *CD274* that were associated with the immune response, were primarily enriched in carcinogenesis pathways and should be further validated as potential anti-cancer targets.

Last but not least, Thanmalagan et al. attempted to explain the biological profile of the disease and to improve the diagnostic and therapeutic tools by analyzing the microarray profile dataset by Harvell et al. (14, 19). In this case, the researchers thoroughly studied the post-translational modification (PTMs) pattern of the DEGs in PABC patients in comparison to non-PABC cases. The researchers evaluated multiple up-regulated and down-regulated DEGs, appraised their corresponding PTMs (phosphorylation, ubiquitylation etc.) and proved that four particular genes (*KLF1, FEN1, SP110, MUC1*) may be recognized as promising therapeutic targets ensuring no harm to pregnancy progress and fetal development.

Among the limitations of this review, it should be stressed that our conclusions are based on studies that utilized heterogeneous genomic approaches (tissue or bioinformatic analysis), different sample preparation methods and sample types (FFPE, fresh frozen tissue). Additionally, no correlation among the genomic profile and the clinicopathological characteristics of PABC was examined in the majority of the studies included in our review. Furthermore, the number of eligible articles was limited due to the rarity of the disease. Thus, we are not allowed to draw definite conclusions and formulate recommendations; further studies should be conducted to confirm the abovementioned observations.

## Conclusion

In conclusion, several studies on PABC indicate an adverse prognostic outcome for the disease that is correlated with its unexplained molecular nature. Highlighting the genomic background of PABC and analyzing all the biological pathways will further facilitate the identification of novel biomarkers defining women among the general population who are at high-risk of developing PABC. Our review systematically summarizes all available data on the distinct genomic profile of PABC offering valuable insight into PABC biological characteristics; this approach may eventually serve as a significant resource for further research in the field and elucidate PABC underlying mechanisms for the disclosure of new diagnostic, prognostic and therapeutic targets. Further research in the field of pregnancy-associated breast cancer is highly recommended as its rate is expected to increase substantially in the upcoming years.

## Data Availability Statement

All datasets generated for this study are included in the article/supplementary material.

## Author Contributions

AMK and EZ were the writers of the article. MM, EZ, and AMK performed the literature search and data extraction from all studies examined. FZ and MAD contributed to the conception and design of the study and to the revision of the manuscript. All authors have read and approved the final manuscript.

## Conflict of Interest

The authors declare that the research was conducted in the absence of any commercial or financial relationships that could be construed as a potential conflict of interest.

## Abbreviations

PABC: pregnancy-associated breast cancer
ER: estrogen receptors
PR: progesterone receptors
HER-2: human epidermal growth factor receptor-2
FFPE: formalin-fixed paraffin embedded
CNA: copy number alteration
GPCR: G-protein-coupled receptor
DFS: disease-free survival
DEG: differentially expressed gene
OS: overall survival
PTM: post-translational modification.

## References

[1] Ahmad A. Breast cancer statistics: recent trends. Adv Exp Med Biol. (2019) 1152:1–7. 10.1007/978-3-030-20301-6_1 31456176

[2] Lee GE Mayer EL Partridge A. Prognosis of pregnancy-associated breast cancer. Breast Cancer Res Treat. (2017) 163:417–21. 10.1007/s10549-017-4224-6 28365832

[3] Zagouri F Psaltopoulou T Dimitrakakis C Bartsch R Dimopoulos MA. Challenges in managing breast cancer during pregnancy. J Thorac Dis. (2013) 5:S62–7. 10.3978/j.issn.2072-1439.2013.05.21 23819029 PMC3695539

[4] Wang B Yang Y Jiang Z Zhao J Mao Y Liu J. Clinicopathological characteristics, diagnosis, and prognosis of pregnancy-associated breast cancer. Thorac Cancer. (2019) 10:1060–8. 10.1111/1759-7714.13045 30920126 PMC6500985

[5] Gooch JC Chun J Kaplowitz E Guth A Axelrod D Shapiro R. Pregnancy-associated breast cancer in a contemporary cohort of newly diagnosed women. Breast J. (2020) 26:668–71. 10.1111/tbj.13510 31448522

[6] Zagouri F Dimitrakakis C Marinopoulos S Tsigginou A Dimopoulos MA. Cancer in pregnancy: disentangling treatment modalities. ESMO Open. (2016) 1:e000016. 10.1136/esmoopen-2015-000016 27843602 PMC5070264

[7] De Haan J Verheecke M Van Calsteren K Van Calster B Shmakov RG Gziri MM. Oncological management and obstetric and neonatal outcomes for women diagnosed with cancer during pregnancy: a 20-year international cohort study of 1170 patients. Lancet Oncol. (2018) 19:337–46. 10.1016/S1470-2045(18)30059-7 29395867

[8] Zanetti-Dällenbach R Tschudin S Lapaire O Holzgreve W Wight E Bitzer J. Psychological management of pregnancy-related breast cancer. Breast. (2006) 15:S53–9. 10.1016/S0960-9776(07)70019-X 17382864

[9] Reed W Sandstad B Holm R Nesland JM. The prognostic impact of hormone receptors and c-erbB-2 in pregnancy-associated breast cancer and their correlation with BRCA1 and cell cycle modulators. Int J Surg Pathol. (2003) 11:65–74. 10.1177/106689690301100201 12754622

[10] Liberati A Altman DG Tetzlaff J Mulrow C Gotzsche PC Ioannidis JP. The PRISMA statement for reporting systematic reviews and meta-analyses of studies that evaluate health care interventions: explanation and elaboration. J Clin Epidemiol. (2009) 62:e1–e34. 10.1016/j.jclinepi.2009.06.006 19631507

[11] Nguyen B Venet D Azim HA Jr Brown D Desmedt C Lambertini M. Breast cancer diagnosed during pregnancy is associated with enrichment of non-silent mutations, mismatch repair deficiency signature and mucin mutations. NPJ Breast Cancer. (2018) 4:23. 10.1038/s41523-018-0077-3 30109263 PMC6078984

[12] Azim HA Jr Brohée S Peccatori FA Desmedt C Loi S Lambrechts D. Biology of breast cancer during pregnancy using genomic profiling. Endocr Relat Cancer. (2014) 21:545–54. 10.1530/ERC-14-0111 24825746

[13] Shen T Vortmeyer AO Zhuang Z Tavassoli F. High frequency of allelic loss of BRCA2 gene in pregnancy-associated breast carcinoma. J Natl Cancer Inst. (1999) 91:1686–7. 10.1093/jnci/91.19.1686 10511599

[14] Harvell DM Kim J O'Brien J Tan AC Borges VF Schedin P. Genomic signatures of pregnancy-associated breast cancer epithelia and stroma and their regulation by estrogens and progesterone. Hor Cancer. (2013) 4:140–53. 10.1007/s12672-013-0136-z 23479404 PMC3810166

[15] Zhang J Zhou YJ Yu ZH Chen AX Yu Y Wang X. Identification of core genes and clinical roles in pregnancy-associated breast cancer based on integrated analysis of different microarray profile datasets. Biosci Rep. (2019) 39:BSR20190019. 10.1042/BSR20190019 31171715 PMC6591572

[16] Zhou Q Sun E Ling L Liu X Zhang M Yin H. Bioinformatic analysis of computational identified differentially expressed genes in tumor stoma of pregnancy-associated breast cancer. Mol Med Rep. (2017) 16:3345–50. 10.3892/mmr.2017.6947 28713995

[17] Johansson O Loman N Borg A Olsson H Pregnancy-associated breast cancer in BRCA1 and BRCA2 germline mutation carriers. Lancet. (1998) 352:1359–60. 10.1016/S0140-6736(05)60750-7 9802282

[18] Walter BA Gómez-Macias G Valera VA Sobel M Merino MJ miR-21 expression in pregnancy-associated breast cancer: a possible marker or poor prognosis. J Cancer. (2011) 2:67–75. 10.7150/jca.2.67 21326627 PMC3039223

[19] Thanmalagan RR Naorem LD Venkatesan A. Expression data analysis for the identification of potential biomarker of pregnancy associated breast cancer. Pathol Oncol Res. (2017) 23:537–44. 10.1007/s12253-016-0133-y 27832451

[20] Mukhopadhyay P Chakraborty S Ponnusamy M Lakshmanan I Jain M Batra SK. Mucins in the pathogenesis of breast cancer: implicationsin diagnosis, prognosis and therapy. Biochim Biophys Acta. (2011) 1815:224–40. 10.1016/j.bbcan.2011.01.001 21277939 PMC3230300

[21] Alexandrov LB Nik-Zainal S Wedge DC Aparicio SAJR Behjati S Biankin AV. Signatures of mutational processes in human cancer. Nature. (2013) 500:415–21. 10.1038/nature12477 23945592 PMC3776390

[22] Dorsam RT Gutkind JS. G-protein-coupled receptors and cancer. Nat Rev Cancer. (2007) 7:79–94. 10.1038/nrc2069 17251915

[23] Pai VP Marshall AM Hernandez LL Buckley AR Horseman ND. Altered serotonin physiology in human breast cancers favors paradoxical growth and cell survival. Breast Cancer Res. (2009) 11:R81. 10.1186/bcr2448 19903352 PMC2815543

[24] Ma XJ Dahiya S Richardson E Erlander M Sgroi DC. Gene expression profiling of the tumor microenvironment during breast cancer progression. Breast Cancer Res. (2009) 11:R7. 10.1186/bcr2222 19187537 PMC2687710

[25] McCready J Arendt LM Rudnick JA Kuperwasser C. The contribution of dynamic stromal remodeling during mammary development to breast carcinogenesis. Breast Cancer Res. (2010) 12:205. 10.1186/bcr2578 20584344 PMC2917019

[26] Rønnov-Jessen L Petersen OW Bissell MJ. Cellular changes involved in conversion of normal to malignant breast: importance of the stromal reaction. Physiol Rev. (1996) 76:69–125. 10.1152/physrev.1996.76.1.69 8592733

[27] Wilcox A Katsanakis KD Bheda F Pillay TS. ASB6, an adipocyte-specific ankyrin and SOCS box protein, interacts with APS to enable recruitment of elongins B and C to the insulin receptor signaling complex. J Biol Chem. (2004) 279:38881–8. 10.1074/jbc.M406101200 15231829

